# Temporal stability of lipid-shelled microbubbles during acoustically-mediated blood-brain barrier opening

**DOI:** 10.3389/fphy.2020.00137

**Published:** 2020-05-06

**Authors:** Antonios N. Pouliopoulos, Daniella A. Jimenez, Alexander Frank, Alexander Robertson, Lin Zhang, Alina R. Kline-Schoder, Vividha Bhaskar, Mitra Harpale, Elizabeth Caso, Nicholas Papapanou, Rachel Anderson, Rachel Li, Elisa E. Konofagou

**Affiliations:** 1. Department of Biomedical Engineering, Columbia University, New York City, New York 10032, USA; 2. Department of Radiology, Columbia University, New York City, New York 10032, USA

**Keywords:** focused ultrasound, microbubbles, temporal stability, contrast agents, passive cavitation detection, blood-brain barrier

## Abstract

Non-invasive blood-brain barrier (BBB) opening using focused ultrasound (FUS) is being tested as a means to locally deliver drugs into the brain. Such FUS therapies require injection of preformed microbubbles, currently used as contrast agents in ultrasound imaging. Although their behavior during exposure to imaging sequences has been well described, our understanding of microbubble stability within a therapeutic field is still not complete. Here, we study the temporal stability of lipid-shelled microbubbles during therapeutic FUS exposure in two timescales: the short time scale (i.e., μs of low-frequency ultrasound exposure) and the long time scale (i.e., days post-activation). We first simulated the microbubble response to low-frequency sonication, and found a strong correlation between viscosity and fragmentation pressure. Activated microbubbles had a concentration decay constant of 0.02 d^−1^ but maintained a quasi-stable size distribution for up to 3 weeks (< 10% variation). Microbubbles flowing through a 4-mm vessel within a tissue-mimicking phantom (5% gelatin) were exposed to therapeutic pulses (f_c_: 0.5 MHz, peak-negative pressure: 300 kPa, pulse length: 1 ms, pulse repetition frequency: 1 Hz, n=10). We recorded and analyzed their acoustic emissions, focusing on emitted energy and its temporal evolution, alongside the frequency content. Measurements were repeated with concentration-matched samples (10^7^ microbubbles/ml) on day 0, 7, 14, and 21 after activation. Temporal stability decreased while inertial cavitation response increased with storage time both *in vitro* and *in vivo*, possibly due to changes in the shell lipid content. Using the same parameters and timepoints, we performed BBB opening in a mouse model (n=3). BBB opening volume measured through T1-weighted contrast-enhanced MRI was equal to 19.1 ± 7.1 mm^3^, 21.8 ± 14 mm^3^, 29.3 ± 2.5 mm^3^, and 38 ± 20.1 mm^3^ on day 0, 7, 14, and 21, respectively, showing no significant difference over time (p-value: 0.49). Contrast enhancement was 24.9 ± 1.7 %, 23.7 ± 11.7 %, 28.9 ± 5.3 %, and 35 ± 13.4 %, respectively (p-value: 0.63). In conclusion, the in-house made microbubbles studied here maintain their capacity to produce similar therapeutic effects over a period of 3 weeks after activation, as long as the natural concentration decay is accounted for. Future work should focus on stability of commercially available microbubbles and tailoring microbubble shell properties towards therapeutic applications.

## INTRODUCTION

Focused ultrasound (FUS) in combination with intravenous injection of microbubbles (MBs) can be used to non-invasively, locally, and reversibly open the blood-brain barrier (BBB) [1,2]. MBs disperse throughout the vasculature and begin vibrating when exposed to the alternating phases of the FUS wave, undergoing a complex set of behaviors termed acoustic cavitation [3]. Intravascular stresses exerted by these vibrations allow for the temporary permeabilization of the otherwise impenetrable BBB. Although a lot of efforts have focused on the development of FUS systems able to perform targeted therapies [4–7], the behavior of MBs exposed to therapeutic FUS has been relatively understudied.

MBs were originally designed and are routinely used as contrast agents in ultrasound imaging applications [8]. As such, their behavior under exposure to center frequencies and pulse lengths relevant to ultrasound imaging has been well described [9,10]. MB lipid shell composition significantly affects the acoustic dissolution rate, fragmentation threshold, and lipid shedding during ultrasound imaging [11]. MB behavior during such sequences is dominated by surfactant shedding during the on-time of μs-long pulses and by gas diffusion during the off-time in kHz pulse repetition frequencies (PRF) [12]. Gas diffusion and stability within circulation can be modified through the addition of poly-ethylene glycol (PEG) in the constituent phospholipids. The degree and type of PEG-ylation had a limited effect on the circulation time and echogenicity of lipid-shelled MBs [13]. In contrast, the lipid molar ratio had a significant effect in the backscattered power, most likely due to different shell viscosity [14]. There is evidence that a decrease in the molar content of PEG-ylated emulsifier increases the shell stiffness [15,16]. Finally, viscosity and stiffness decrease with temperature elevation, unlike the size distribution which remains largely unaffected [17].

In the therapeutic ultrasound realm, most previous work has focused on drug-loaded MBs [18]. In terms of brain therapy, it has been shown that the MB type [19] and size distribution [20] are defining factors in BBB opening efficiency. Size-isolated MBs [21] with larger average diameters produced larger BBB opening volumes [22], due to enhanced engagement with the surrounding microvasculature [23]. In terms of the physicochemical properties of the MB shell, longer hydrophobic chains in the phospholipid layer led to increased acoustic emissions and drug delivery, especially at high acoustic pressures [24]. Heavy gas cores are required to avoid fast dissolution through the lipid shell, but the gas type does not appear to significantly influence the BBB opening efficiency [25].

Therapeutic pulses differ from imaging pulses in terms of their center frequency and pulse length. Low-frequency (< 1.5 MHz) and ms-long (> 500 cycles) pulses are typically used for BBB opening and targeted drug delivery applications [26–29]. Such pulses promote primary [30,31] and secondary [32,33] Bjerknes forces, lower the inertial cavitation threshold [34,35], favor coalescence [36,37] and produce sustained acoustic streaming within the blood vessels [38–40]. All these effects are expected to influence the stability of MBs during therapeutic ultrasound exposure, and in turn, the resulting bioeffect [41]. Low-frequency insonation leads to significantly higher MB expansion ratios compared to imaging center frequencies [42]. MB stability during therapeutic ultrasound exposure depends on the characteristics of the ultrasound pulse sequence used [43,44]. Short pulses emitted at PRFs on the order of kHz prolong the MB lifetime [43], improve the spatiotemporal uniformity of cavitation activity [44], and eliminate standing-wave formation within the skull [45,46]. Enhanced temporal stability along with uniform cavitation activity have produced uniform and minute-lasting BBB opening [47,48]. All these studies were conducted with a specified MB formulation and shed light on the influence of the exposure conditions to the MB stability.

To date, there has been no study to investigate the temporal stability of MBs with variable phospholipid molar ratios in ultrasound therapy, and in particular in the context of BBB opening. Furthermore, an important parameter which may be useful in both pre-clinical and clinical investigations is the stability of MBs during therapeutic exposure after long-term storage following activation. One study examining Definity® MB stability over a period of 15 days post-activation found a large variation in the MB collapse threshold, which did not follow a linear trend over time [49]. Size-isolated MBs had stable size distributions over time for up to a month post-activation [21]. Here, we studied temporal stability of polydisperse lipid-shelled MBs in two time scales: a) short time scale, i.e. μs of therapeutic ultrasound exposure, and b) long time scale, i.e. days post-activation. We conducted numerical simulations, *in vitro* phantom experiments and *in vivo* BBB opening in a mouse model, in order to establish the characteristics of the acoustic emissions over these two time scales. Our hypothesis was that the lipid molar ratio and storage time do not change the temporal stability and the BBB opening potential of lipid-shelled MBs.

## METHODS

### Numerical simulations

To evaluate the effect of shell parameters on the MB oscillation dynamics, and more importantly the break-up or fragmentation pressure, we implemented the Marmottant model [50] in MATLAB^©^ (The Mathworks, Natick, MA, USA). This model is based on the Rayleigh-Plesset equation, modified to include the effect of the shell characteristics [50]:

(1) $$\rho_{l}\left(R\ddot{R}+\frac{3}{2}{\dot{R}}^{2}\right)=\left[P_{0}+\frac{2\sigma \left(R_{0}\right)}{R_{0}}\right]{\left(\frac{R}{R_{0}}\right)}^{-3\kappa }\left(1-\frac{3\kappa }{c}\dot{R}\right)-P_{0}-\frac{2\sigma (R)}{R}-\frac{4\mu \dot{R}}{R}-\frac{4\kappa_{S}\dot{R}}{R^{2}}-P(t)$$

with surface tension *σ*(*R*) being:

(2) $$\sigma (R)=\{\begin{array}{l} 0\text{ if }R\leq R_{\text{buckling }}\\ \chi \left(\frac{R^{2}}{R_{\text{buckling }}^{2}}-1\right)\text{ if }R_{\text{buckling }}\leq R\leq R_{\text{break-up }}\\ \sigma_{\text{water }}\text{ if }R>R_{\text{break-up }} \end{array}.$$

All parameters used here were based on reported literature (table 1). Shell compression modulus *χ* and surface dilatational viscosity *κ*_*S*_ were estimated based on previous work [14], assuming a linear increase of both with increasing molar ratio. Lipid layer elasticity has been shown to increase with a reduction of the DSPE-PEG2000 content [15,16], or conversely, an increase in the DSPC:DSPE-PEG2000 molar ratio in this study. Similar observations have been made regarding shell viscosity [14,51]. Yet, the elasticity and viscosity increase with molar ratio is an assumption and may influence the validity of the simulations. Furthermore, we assumed a thin lipid shell of thickness ε equal to 1 nm [51]. *χ* and *κ*_*S*_ of the thin lipid shell were calculated by multiplying the relative bulk moduli with the shell thickness, i.e. *χ* = 3*G*_*s*_*ε* and *κ*_*S*_ =3*μ*_*lipid*_*ε* [50,52], where *G*_*s*_ and *μ*_*lipid*_*ε* were the bulk shear modulus and the bulk viscosity of the lipids constituting the shell [14]. Marmottant model assumes that *ε*≪*R* , which is generally true for lipid-shelled MBs. However, *ε* can be up to 650 nm in polymer-shelled MBs [53]. In this study, the buckling radius was assumed to be equal to the equilibrium radius (i.e., 1.2 μm). Equation (1) was solved using the built-in *ode45* solver in MATLAB^©^, a fourth-order Runge-Kutta algorithm, with a time step of 10 ns. This time step was identical to the sampling period used in the *in vitro* and *in vivo* experiments, to allow for meaningful comparison.

### Microbubble formulation

**FIGURE 1 |:**
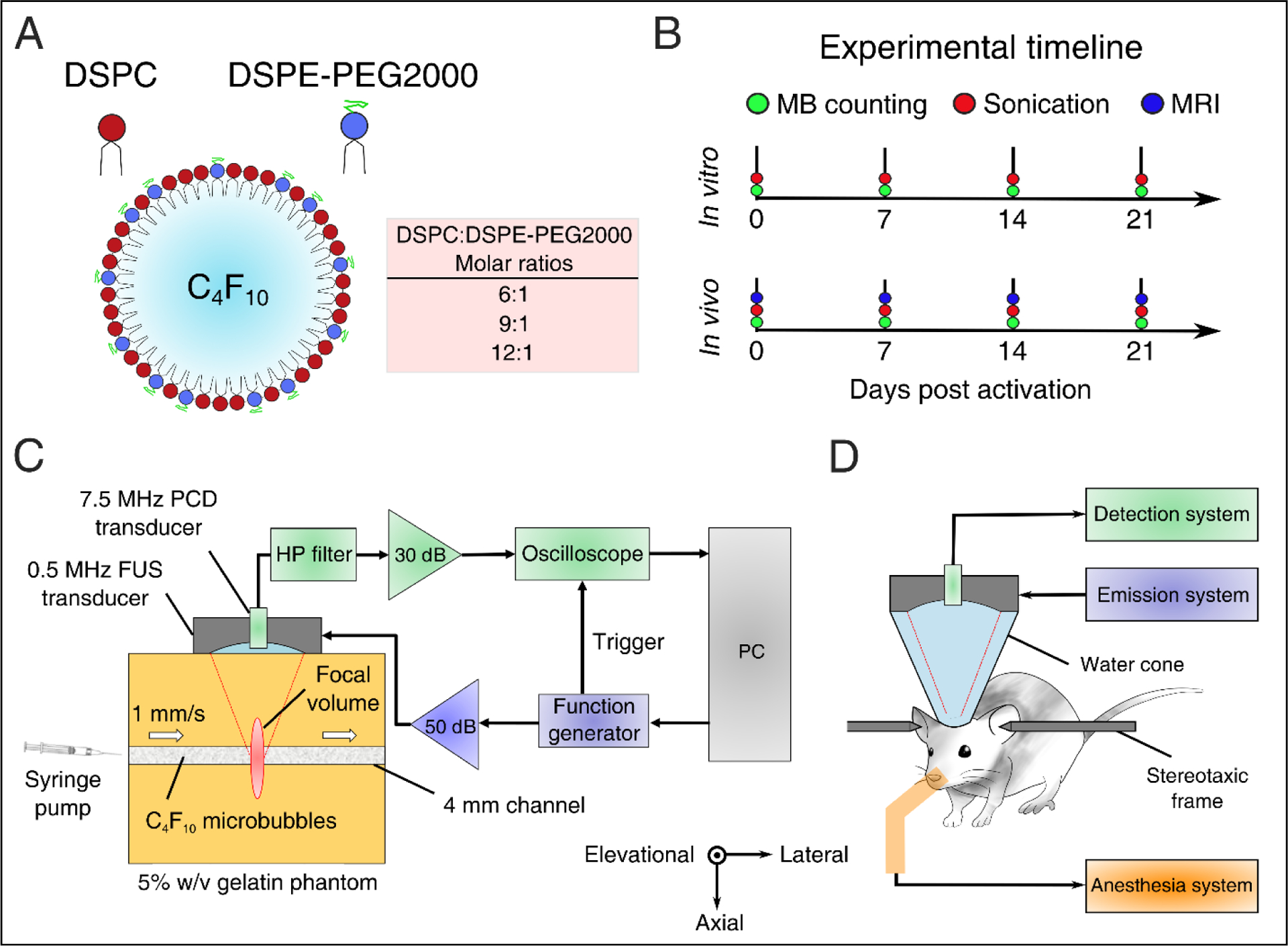
Experimental outline and setups. (A) Microbubble formulation. DSPC and DSPE-PEG2000 were mixed at different lipid molar ratios to produce microbubbles of variable shell stiffness and viscosity. (B) Experimental timeline for estimating microbubble stability *in vitro* and *in vivo*. (C) *In vitro* experimental setup using a 5% w/v tissue-mimicking phantom. (D) *In vivo* experimental setup for non-invasive blood-brain barrier opening in mice. Abbreviations: FUS – focused ultrasound; PCD – passive cavitation detection; HP – 1.2-MHz high-pass.

Lipid-shelled MBs were prepared in-house following previously described chemical synthesis protocols [21,54]. Briefly, the shell constituted of two lipids, 1,2-distearoyl-sn-glycero-3-phosphocholine (DSPC) and 1,2-distearoyl-sn-glycero-3-phosphoethanolamine-N-[methoxy(polyethylene glycol)-2000] (DSPE-PEG2000 or DSPE-PEG2K hereafter) (No. 850365 and 880120, purity > 99%; Avanti Polar Lipids, Alabaster, AL, USA) mixed at variable molar ratios (6:1, 9:1, and 12:1 - or in percentage format, 86:14, 90:10, and 92:8; Figure 1A). *In vivo* experiments and most *in vitro* experiments were conducted with the 9:1 molar ratio, which is typically used for BBB opening [20] and corresponds to a Definity-like mixture [55]. The ratios of 6:1 and 12:1 were selected on either side of the established ratio, to investigate the effect of using less or more emulsifier on the cavitation response. Lipids were mixed within a solution of 80% v/v PBS, 10% v/v glycerol, and 10% v/v 1,2-propanediol (Sigma Aldrich, St. Louis, MI, USA). Perfluorobutane (C_4_F_10_; FluoroMed LP, Round Rock, TX, USA) was introduced in the empty head space of the hosting vial, and was then mechanically mixed with the lipid solution using an amalgamator for 45 s (Vialmix; Lantheus Imaging, North Billerica, MA, USA). MB activation was performed on day 0, but MBs were counted and sized prior to every experiment (Figure 1B), in order to have concentration-matched populations for each sonication. MBs were stored in room temperature to avoid large temperature gradients during the course of the experiments, which could influence the size distribution or shell properties [17]. Following activation, MB vials were covered with parafilm to reduce the amount of gas exchange between the vial and the environment. Yet, nitrogen and oxygen transfer into the PFB core is likely to affect the stability and inertial cavitation responses over time [56].

### Experimental setup

*In vitro* and *in vivo* experiments were conducted in the same experimental setup (Figures 1C and 1D), described in detail elsewhere [57]. Briefly, a 0.5-MHz spherical-segment single-element FUS transducer (Part No. H-204; Sonic Concepts, Bothell, WA, USA) was driven by a waveform generator (33500B series; Agilent technologies, Santa Clara, CA, USA) through a 50-dB radiofrequency power amplifier (Model A075; E&I, Rochester, NY, USA). The focal volume (2 mm × 11 mm) was placed either at the center of the 4-mm channel of the tissue-mimicking gelatin phantom (concentration: 5% w/v) or at the caudate putamen structure of the murine brain. For the *in vitro* experiment, MBs were flowing through the channel at a constant velocity of 1 mm/s, to imitate the slow flow of capillaries. Acoustic emissions were captured with a 7.5-MHz single-element passive cavitation detector (Part No. U8423539, V320, diameter: 12.7 mm, focal depth: 76.2 mm; Olympus Industrial, Waltham, MA, USA) which was inserted and co-aligned with FUS transducer, having overlapping foci. A high-pass filter was used to filter out the fundamental and the second harmonic reflections (Part No. ZFHP-1R2-S+, cut-off frequency 1.2 MHz; Mini Circuits, Brooklyn, NY, USA). Recorded signals were amplified by 30-dB with a pulser-receiver (Part No. 5072; Olympus Industrial) and then recorded using a GaGe oscilloscope card (Part No. CSE1422, 14 bit; Dynamic Signals LLC, Lockport, IL, USA). We captured segments of 114,688 time points at a sampling frequency of 100 MSa/s.

### Signal processing

**FIGURE 2 |:**
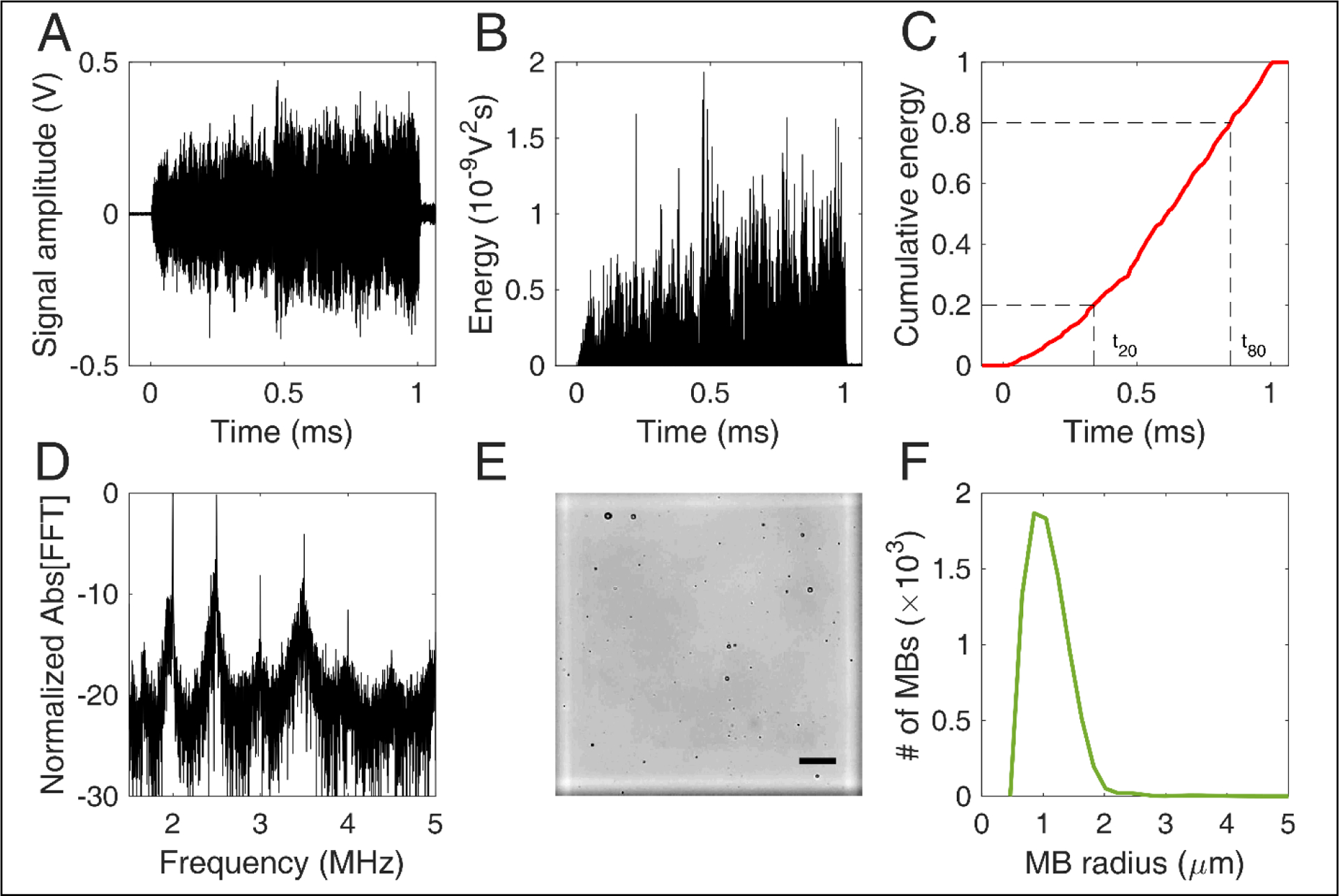
Signal and image processing. (A) Time-domain signal capturing cavitation emissions during the 1-ms-long therapeutic pulse. B) Energy evolution during a single pulse. (C) Normalized cumulative energy during a single pulse. Time constants t_20_ and t_80_ were defined as the time required for 20% and 80% of the total acoustic energy to be emitted, respectively. D) Normalized amplitude of fast Fourier Transform (FFT) performed over the cavitation emissions produced by a single pulse. (E) Example of an optical microscopy image acquired for microbubble counting and sizing. The marked square of the hemocytometer is in white. Scale bar: 50 μm. (F) Microbubble size distribution estimated through optical microscopy.

Acoustic cavitation emissions were processed offline in MATLAB^©^. Time-domain signal (Figure 2A) was used to estimate the energy (Figure 2B) emitted during a single therapeutic pulse through:

(3) $$E\sim \int_{0}^{T}V^{2}dt\approx \sum_{t=0}^{T}V^{2}\Delta t$$

where *V* was the voltage at each time point in volts and Δ*t* was the sampling period equal to 10 ns or 10^−8^ s. In this calculation, it was assumed that the electrical energy in the detection system was proportional to the acoustic energy emitted by the MBs. We also assumed a dimensionless resistance value of 1 for simplicity, therefore energy units are given in V^2^s and not in Joule. Control sonications without MBs were used to estimate the baseline signal [58], whose energy was subtracted from the MB acoustic emissions at each time point [43]. We then assessed the normalized cumulative energy (Figure 2C) to investigate the temporal distribution of cavitation emissions during the pulse. To do so, two temporal constants were calculated at each condition, following previous work [43,44]. The constants t_20_ and t_80_ were defined as the time required for the 20% and 80% of the total acoustic energy to be emitted (Figure 2C). Based on these two values, a third stability metric was introduced, namely temporal bias (TB). TB was defined as:

(4) $$TB=t_{80}/t_{20}-4.$$

TB equal to 0 would indicate a symmetric profile of emissions over time, since for “linear emission curves” t_80_ would be four times t_20_. Negative TB values (i.e., t_80_ < 4t_20_) would indicate early emission bias, while positive TB values (i.e., t_80_ > 4t_20_) would indicate late emission bias.

Frequency analysis was conducted to identify the cavitation mode at each experimental condition. A Fast Fourier transform (FFT) was performed in MATLAB (number of FFT points: 114,688). Based on the FFT (Figure 2D), three spectral areas were filtered and analyzed independently:

(5) $$\text{a})\;\text{harmonic}\;\text{regions,}\;f_{h,n}\;=\;nf_{c}$$

(6) $$\text{b})\;\text{ultraharmonic}\;\text{regions,}\;f_{u,n}\;=\;\left(n-1/2\right)f_{c}$$

(7) $$\text{c})\;\text{broadband}\;\text{regions}\;f_{b},\;\text{With}\;f_{h,n}\;+10kHz<f_{b}<f_{u,n}-10kHz\;\text{and}\;f_{u,n}+10kHz<f_{b}<f_{h,n+1}-10kHz.$$

where *f*_*c*_ was the center frequency of the FUS transducer (i.e., 0.5 MHz) and *n* was the harmonic number (*n* = 3,4,5,…,10). The fundamental and second harmonics were filtered out and ignored, due to strong reflections at these frequencies in control experiments.

Cavitation doses were calculated as described before [7,59], based on the root-mean-square voltage detected in the respective spectral areas. Harmonic stable (SCD_h_), ultraharmonic stable (SCD_u_) and inertial cavitation (ICD) doses were defined as:

(8) $$CD_{i}=\sqrt{{\langle |FFT{|}_{f_{i}}^{2}\rangle }_{n}}$$

where the index *i* changed for harmonic, ultraharmonic, and broadband regions *f*_*i*_ , to estimate SCD_h_, SCD_u_, and ICD, respectively. These doses were calculated for each acoustic pulse both for *in vitro* and *in vivo* experiments. Wherever appropriate, per-pulse cavitation doses (i.e., cavitation levels) were either averaged or summed to derive the mean and total cavitation doses.

### Image processing for MB sizing

To estimate the MB size distribution, we followed an optical microscopy-based technique similar to previously described approaches [60–62]. Activated MBs were first diluted by 1,000× in distilled water. Ten microliters of this solution were then injected into the chambers at either side of a disposable hemocytometer (part number: NC0435502; Fisher Scientific, Hampton, NH, USA). Each chamber had a height of 100 μm, so the total volume of each marked square was 0.1 mm^3^ (Figure 2E). MBs were imaged in bright field at 20× magnification in an upright microscope (Leica DM6 B; Leica Microsystems Inc., Buffalo Grove, IL, USA). A total of 64 images were acquired, one for each marked square. The images were then cropped, removing the dark rim surrounding the squares of the hemocytometer. Cropped images were processed in Matlab (The Mathworks, Natick, MA, USA) using a purpose-built algorithm that detected individual MBs based on the circular Hough transform (function *imfindcircles*). Given the known volume, the total number of MBs allowed an approximation of the original MB concentration. Finally, the mean and maximum radius of each MB population was calculated at each time point. Different MB batches were used for *in vitro* and *in vivo* experiments, and each batch was measured separately. The same MB batch was used across time points, following activation on day 0.

### In vitro *experiments*

A tissue-mimicking phantom was prepared for the *in vitro* experiments. Gelatin powder (G2500; Sigma Aldrich, St. Louis, MI, USA) was slowly mixed in hot water (>60 °C), which was continuously stirred with a magnetic stirrer. The final gelatin concentration was 5% w/v. A silicon elastomer tube (outer diameter: 4mm; Saint-Gobain, Wayne, NJ, USA) was fixed between the inlet and outlet ports of a plastic container and served as the mold for the channel. The gelatin solution was poured into the container and left over night at 5 °C to set.

The following day, the FUS transducer was placed on top of the gelatin phantom (Figure 1C). A raster scan was performed to locate the channel along the lateral and elevational dimensions. The focal volume was placed at the center of the channel along the axial dimension, using pulse echo. Control sonications were conducted with water flowing at a velocity of 1 mm/s in order to imitate slow capillary flow. Finally, MBs were diluted at the desired concentration (10^7^ MBs/ml) based on the counting result (Figure 5) and were made to flow at the same fluid velocity. A total of 10 therapeutic pulses (Table 2) were emitted per condition.

### In vivo *experiments*

All animal experiments were approved by the Institutional Animal Care and Use Committee (IACUC) of Columbia University. Three wild-type mice (C57BL/6, age: 4 – 8 months, mass: 28 ± 6 g) were exposed to therapeutic ultrasound on a weekly basis. Based on literature, n = 3 mice would suffice to produce statistically significant differences in terms of MRI-based BBB opening quantification [20,63,64]. Anesthesia was induced and maintained with inhalable isoflurane mixed with oxygen (2–3% for induction and 1.2–1.5% for maintenance), delivered through a digital vaporizer (SomnoSuite; Kent Scientific, Torrington, CT, USA). Mice were fixed within a stereotaxic frame (David Kopf Instruments, Tujunga, CA, USA) to allow for accurate targeting (Figure 1D). Head fur was removed with clippers and depilatory cream, applied for 10–20 sec. Using a previously described metallic grid method [26], we targeted the caudate area (coordinates from lambdoid suture: +3 mm ventral, −2 mm lateral). A control sonication was performed prior to MB injection, to acquire a baseline signal, which was subsequently subtracted from the MB signal. MBs were injected through an intravenous catheter inserted into the tail vein, at a concentration of 10^7^ MBs/ml of blood. This concentration was equivalent to 5× the clinical dose of Definity® MBs recommended for ultrasound imaging applications. For each day during the 3 weeks post activation, the injected dose was calculated based on the concentration measured prior to every experiment (Figure 5C).

Following the 2-min ultrasound treatment using clinically relevant acoustic parameters (Table 2), we injected 200 μl of gadolinium(Gd)-based contrast agent (Omniscan; GE healthcare, Bronx, NY, USA) intraperitoneally. Mice were transferred to the MRI suite, anesthetized with 1–2% isoflurane, placed in a 3-cm birdcage coil and scanned with a small-animal 9.4T MRI system (Bruker, Billerica, MA, USA). A contrast-enhanced T1-weighted 2D FLASH scan (TR/TE: 230/3.3 ms, flip angle: 70°, number of excitations: 18, in-plane resolution: 85μm × 85μm, slice thickness: 500 μm, receiver bandwidth: 50 kHz) was acquired approximately 45 minutes after FUS exposure, along both axial and coronal planes.

### Image processing for MRI quantification

MRI scans were loaded into MATLAB^©^. Quantification was performed on the coronal slices. Firstly, a region of interest (ROI) was defined in the contralateral hemisphere to calculate the baseline intensity. The threshold intensity to define BBB opening was set as the average intensity within the control ROI plus 3 standard deviations. Every coronal slice was loaded sequentially, and a manual ROI was drawn within the entire ipsilateral hemisphere. All pixels having intensity higher than the threshold were counted to derive the BBB opening surface area in each slice. The total BBB opening volume (in mm^3^) per mouse was calculated by summing the BBB opening surface areas across all slices and then multiplying by the slice thickness. Finally, the contrast enhancement (in %) was calculated by dividing the mean intensity within the BBB opening areas with the mean intensity of the control ROI.

### Statistics

*In vitro* experiments were repeated for n = 10 pulses and *in vivo* experiments were repeated for n = 3 mice (or 360 pulses) per day post-activation. Measurements are presented as mean ± standard deviation, unless otherwise stated. One-way ANOVA tests with post-hoc Bonferroni analysis were performed to compare metrics across the lipid molar ratios or days post-activation. Statistical significance was assumed at *p* < 0.05.

## RESULTS AND DISCUSSION

### Numerical simulations

**FIGURE 3 |:**
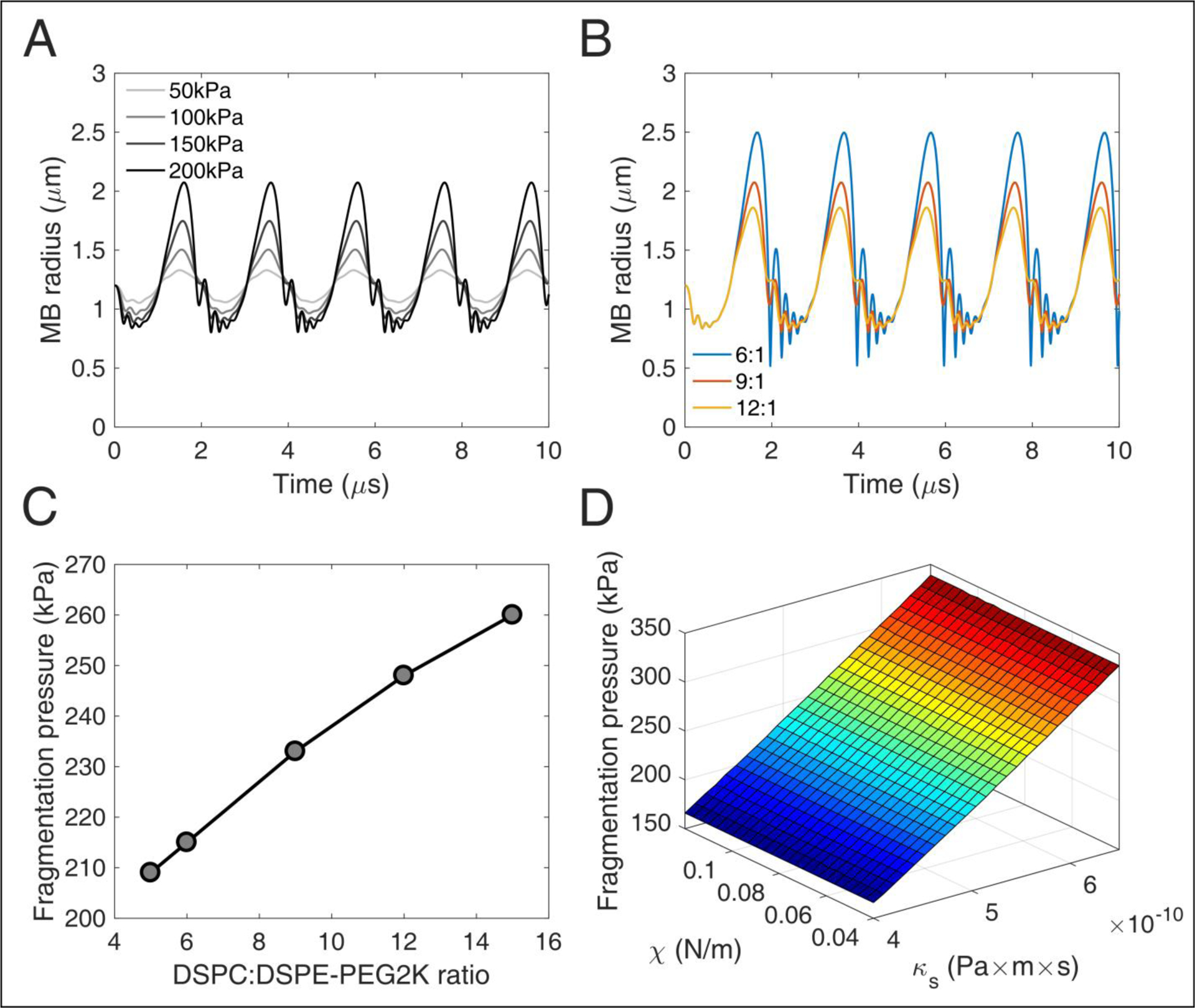
Numerical simulations of microbubble stability. (A) Radius over time for different acoustic pressures. DSPC:**DSPE-PEG2K** ratio: 9:1. (B) Radius over time for different DSPC:**DSPE-PEG2K** ratios. Peak-negative pressure: 200 kPa. (C) Fragmentation pressure across the DSPC:**DSPE-PEG2K** ratios. (D) Fragmentation pressure as a function of compression modulus *χ* and shell dilatational viscosity *κ*_*S*_.

Using equation (1), we simulated the radial oscillations of MBs exposed to therapeutic ultrasound (Figure 3). At low acoustic pressures (e.g., 50 kPa) MBs oscillated in a quasi-sinusoidal fashion around the equilibrium radius. Increasing the acoustic pressure led to asymmetric oscillations, with the expansion phase outweighing the compression phase (Figure 3A). At 200 kPa, the expansion ratio during the rarefactional phase reached up to 1.75 (i.e., maximum radius of 2.1 μm compared to equilibrium radius of 1.2 μm). Additionally, we observed a high-frequency oscillation during the compression phase at high pressures. This effect was more pronounced in MBs with lower DSPC:DSPE-PEG2K molar ratio (Figure 3B). Increasing the molar ratio or, conversely, the shell stiffness and viscosity, suppressed the instability during the MB collapse. Increasing the molar ratio decreased the amplitude of radial oscillations. Consequently, the fragmentation or break-up pressure increased with DSPC:DSPE-PEG2K ratio (Figure 3C). Surface dilatational viscosity had a significant effect on the fragmentation pressure, unlike shell compression modulus (Figure 3D). This is in accordance with previous studies that reported shell viscosity to be the major determinant in MB response [14,51].

### Effect of molar ratio on cavitation response

**FIGURE 4 |:**
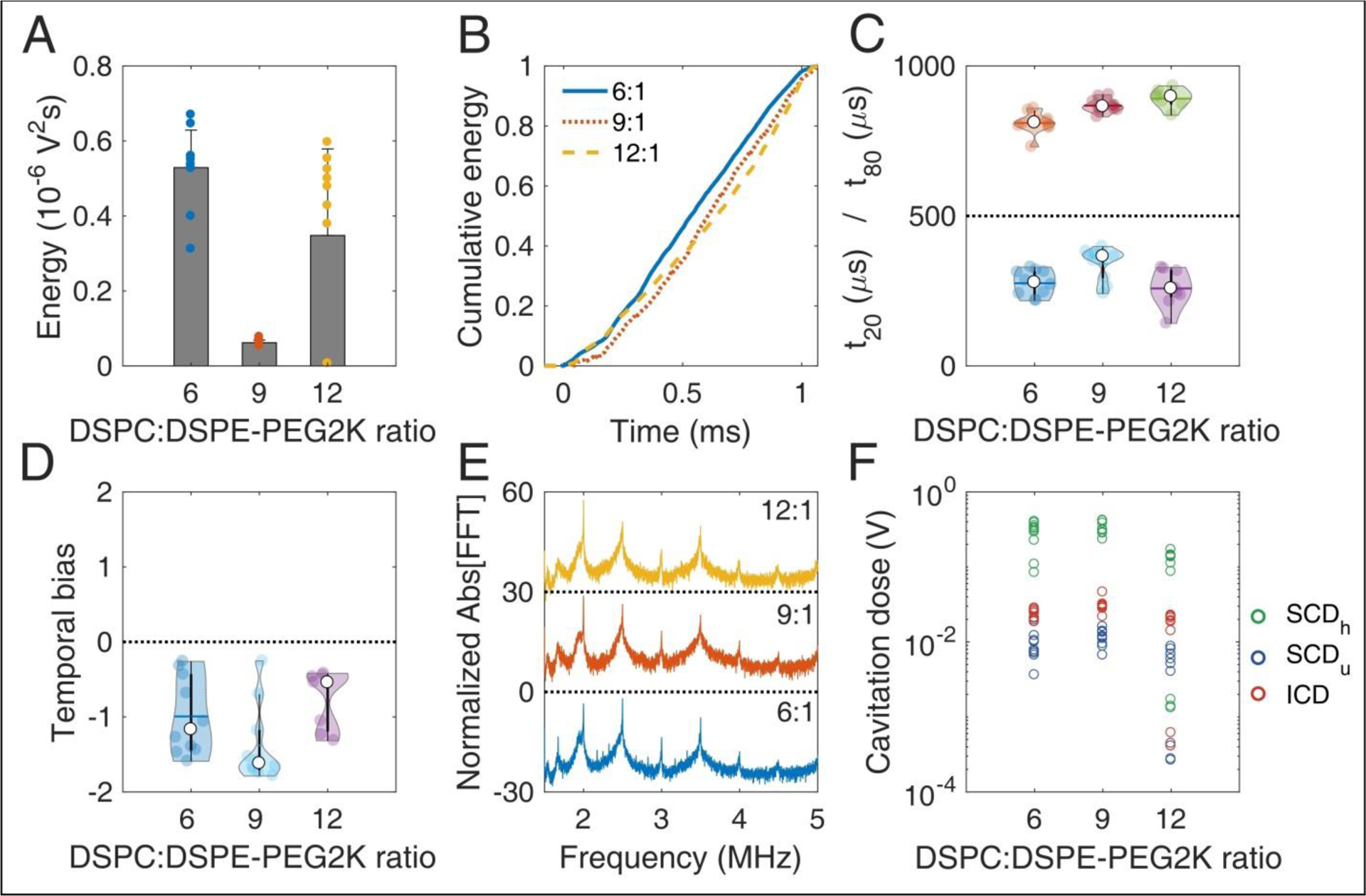
*In vitro* stability of microbubbles encapsulated with lipid shells of variable DSPC:DSPE-PEG2K lipid molar ratios. (A) Total acoustic energy emitted per therapeutic pulse. (B) Mean cumulative energy evolution (n=10) for molar ratios of 6:1 (blue straight line), 9:1 (dotted orange line), and 12:1 (yellow dashed line). (C) Temporal constants t_20_ (t < 500 μs) and t_80_ (t > 500 μs). (D) Temporal bias. (E) Normalized spectra averaged across pulses (n=10). (F) Stable harmonic (green circles), stable ultraharmonic (blue circles), and inertial (red circles) cavitation doses. Peak-negative pressure: 300kPa.

Changing the DSPC:DSPE-PEG2K molar ratio during MB preparation significantly affected most stability metrics (Figure 4). The total energy emitted during sonication was significantly different across the ratios (p = 1.16×10^−6^; one-way ANOVA). The ratio 9:1 produced significantly lower total energy compared to 6:1 and 12:1 (Figure 4A). Qualitatively, the average cumulative energy was similar across the ratios (Figure 4B). However, there were significant differences both in t_20_ (p = 0.005) and t_80_ (p = 2.48×10^−5^). Interestingly, 9:1 ratio had significantly higher t_20_ compared to the other ratios, indicating that early destruction of quasi-resonant MBs is avoided at this ratio (Figure 4C). In contrast, t_80_ increased with molar ratio, suggesting that surviving MBs with higher ratios are more stable compared to lower ratios. This is in accordance with the simulation results, showing that non-resonant bubbles of 1.2 μm in radius are progressively less prone to fragmentation with increasing molar ratio (Figures 2C and 2D).

There was no significant difference in the temporal bias across the molar ratios (p = 0.054; Figure 4D). Average spectra of different ratios had similar features, with a notable increase of both harmonic and broadband peaks for the 9:1 ratio. This was evident in the cavitation doses, where we found a significant difference across ratios, for SCD_h_ (p = 1.87×10^−6^), SCD_u_ (p = 4.92 × 10^−4^) and ICD (p = 4.99 × 10^−5^). 9:1 ratio had higher SCD_h_, SCD_u_, and ICD compared to the other ratios (significantly higher only compared to 12:1). Higher doses indicated sustained acoustic emissions over time, despite the lower total energy emitted (Figure 4A), which may be associated with reduced MB destruction. Taken together, these data indicate that the molar ratio of 9:1 provided the higher stability during the 1-ms pulse, thus it was chosen as the most suitable formulation for therapeutic applications.

### Stability of microbubble size distribution

**FIGURE 5 |:**
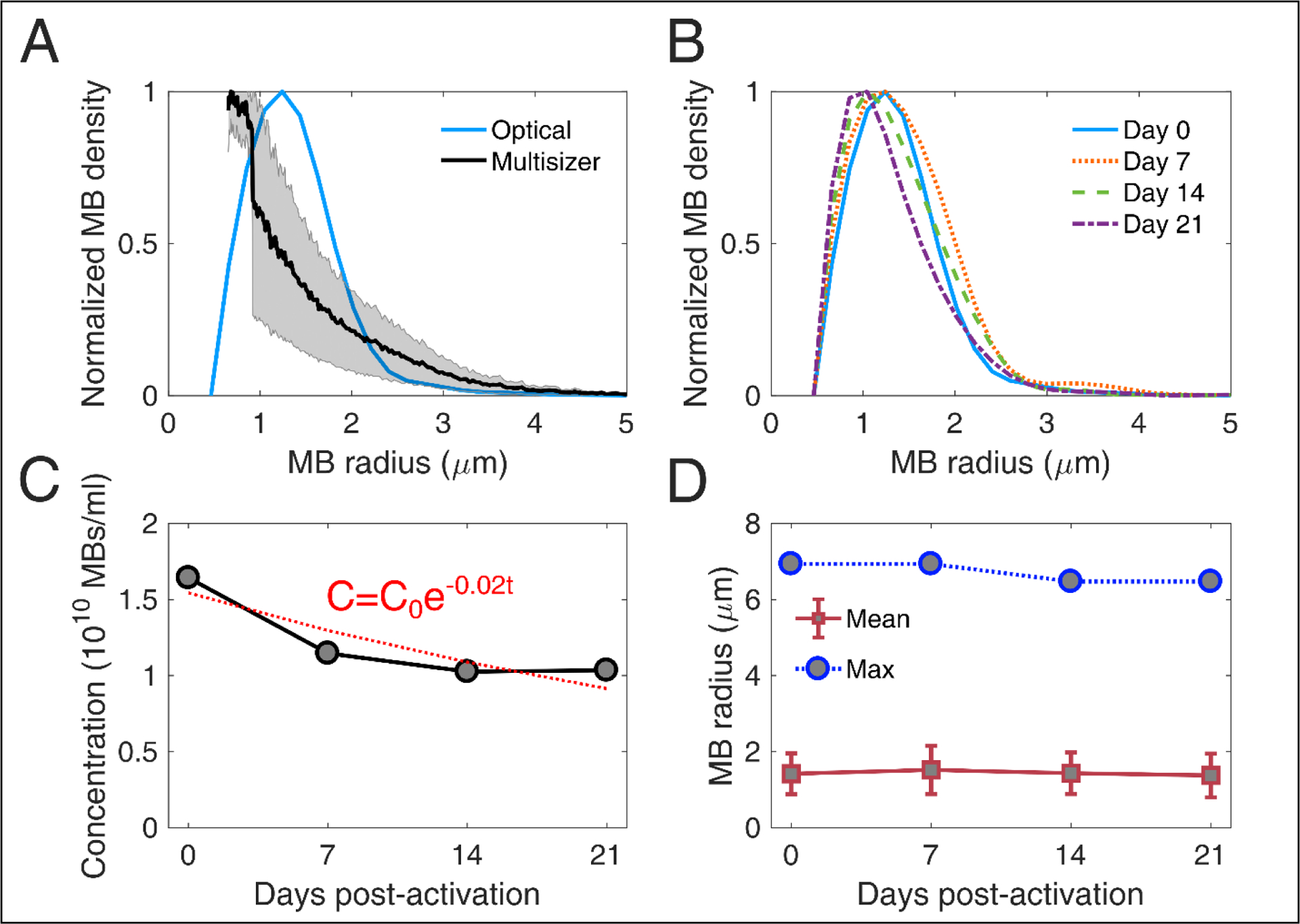
Stability of microbubble size distribution and concentration . (A) Comparison between size distribution estimated through Coulter multisizer (black line; gray area denotes standard deviation, n = 3) and bright field microscopy (blue line). The root mean square error in microbubble density estimation between the two techniques was 0.22 or 22%. (B) Size distribution evolution over time, measured on day 0 (blue straight line), day 7 (dotted orange line), day 14 (dashed green line), and day 21 (dotted-dashed purple line) post-activation. (C) Microbubble concentration over time (grey circles), fitted with an exponential decay curve (red dotted line). The exponential decay factor was estimated at 0.02. C_0_ denotes microbubble concentration on day 0, and t is storage time in days. (D) Evolution of mean (red boxes) and maximum (blue circles) microbubble radius over time. Mean radii are given as mean ± standard deviation.

**FIGURE 6 |:**
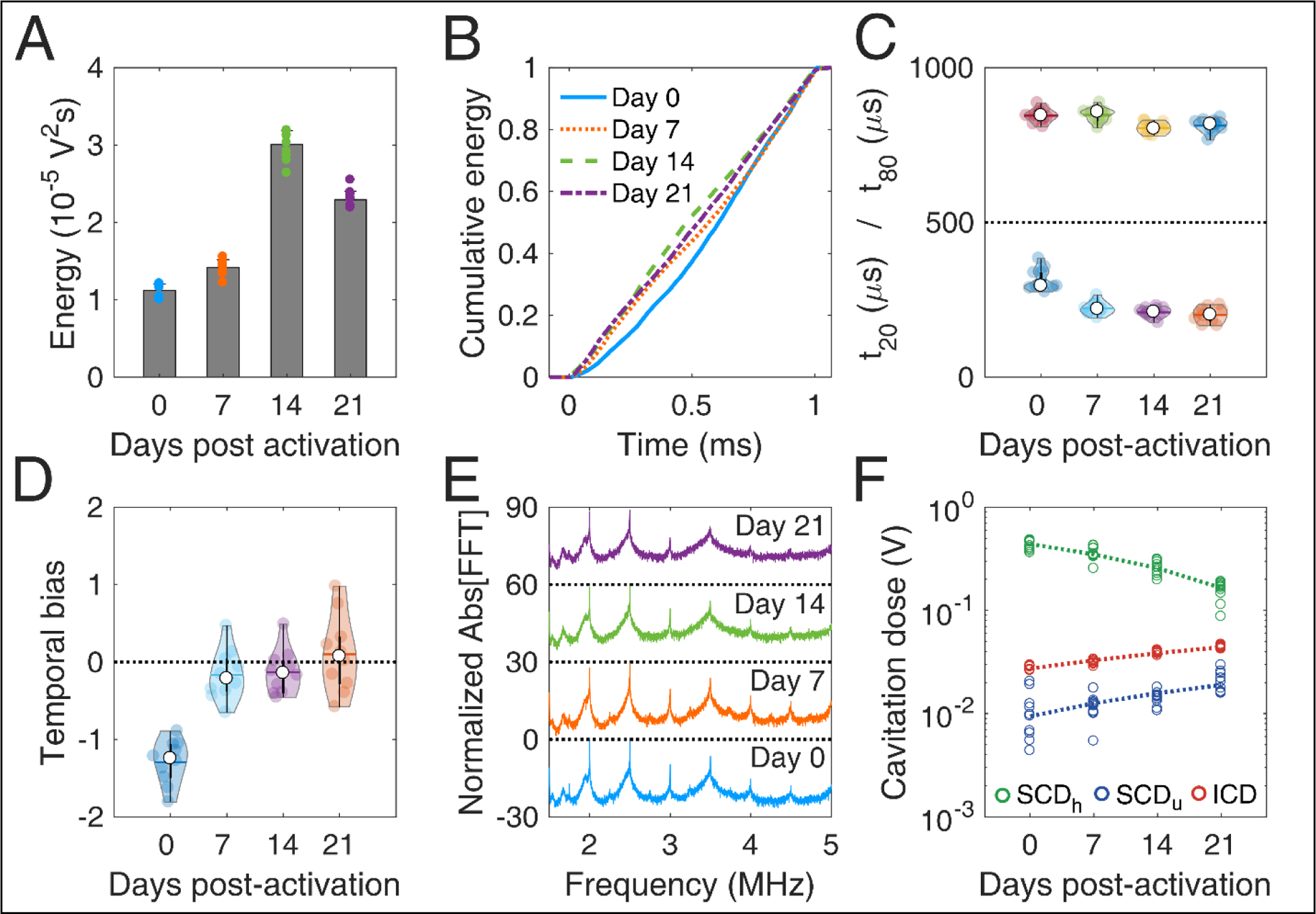
*In vitro* microbubble stability over time. (A) Total acoustic energy emitted per therapeutic pulse over time post-activation. (B) Mean cumulative energy evolution (n=10) for microbubbles exposed to ultrasound on day 0 (blue straight line), day 7 (dotted orange line), day 14 (dashed green line), and day 21 (dotted-dashed purples line) post-activation. (C) Temporal constants t_20_ (t < 500 μs) and t_80_ (t > 500 μs) over time post-activation. (D) Temporal bias over time. (E) Normalized spectra averaged across pulses (n=10). (F) Stable harmonic (green circles), stable ultraharmonic (blue circles), and inertial (red circles) cavitation doses over time. A linear fit was performed on each dose (dashed lines) to investigate the average effect of storage time on cavitation dose.

Long-term storage of activated MBs may be an important factor of reducing cost and allowing widespread use of MB-based FUS therapies, such as BBB opening. For that reason, we first measured the stability of MB size distribution over time (Figure 5). In this study, we used an optical microscopy based counting technique (Figures 2E and 2F). We first compared the size distribution acquired using this method with an alternative technique, based on Multisizer counting (Figure 5A). The derived distributions peaked at different radii (1.24 μm for optical microscopy and 0.75 μm for Multisizer) and had lower degree of agreement in MB radii below 2 μm. Optical microscopy gave a larger MB density for radii between 1 μm and 2 μm compared to Multisizer. The root mean square error in MB density estimation was 0.22 or 22%. Over time, the MB size distributions had similar characteristics (Figure 5B), with a moderate shift of the peak radius towards smaller radii. MB concentration decreased over time (Figure 6C). An exponential fit was performed, assuming that the decay rate was proportional to the remaining MB number. The characteristic decay constant was estimated as *λ* = 0.02*d*^−1^, yielding a MB half-life of 35 days. However, the concentration was practically stable between day 14 and 21 in our measurements. The mean and maximum radius had a limited variation throughout the 3 weeks of measurements (Figure 5D). Mean radius ranged between 1.37 ± 0.56 μm and 1.52 ± 0.63 μm (9.9% variation), while the largest radius measured was between 6.47 μm and 6.94 μm (6.7% variation). This is in accordance with published literature on size-isolated MBs, whose size distribution was stable for up to a month after activation [21].

Previous work has identified discrepancies in the measured size distribution when using different techniques. For example, size distributions were different between Accusizer, which is based on light scattering, and Multisizer, which is based on electrical impedance sensing of displaced electrolyte volume [21,65]. Similarly to this study, it has been previously shown that optical microscopy-based size distribution is not identical to Multisizer measurements [66]. Despite the differences, our main interest was to evaluate the evolution of MB population over time, measured with the same technique (Figure 5D). Critically, the measured concentration (Figure 5C) was used to study the response of concentration-matched samples at every time point, for both *in vitro* and *in vivo* experiments.

### *Microbubble stability* in vitro

MBs with DSPC:DSPE-PEG2K molar ratio of 9:1 were activated on day 0 and were stored in room temperature (~18–20 °C) for 21 days. Concentration-matched MBs were made to flow through the tissue-mimicking phantom (Figure 1C) and were exposed to therapeutic pulses (Table 2), on days 0, 7, 14, and 21. The total emitted energy was significantly different across days (p < 10^−27^), and peaked at day 14 (Figure 6A). Cumulative energy had a smoother slope on day 0 compared to following days (Figure 6B), suggesting slower MB destruction during the first half of the pulse. This was corroborated by the temporal constants. Both t_20_ and t_80_ were significantly different across days (p = 4.5 × 10^−12^ and 5 × 10^−5^, respectively). Both constants progressively decreased over time (Figure 6C), with the effect being stronger on t_20_ (i.e., at the beginning of the pulse). Sonications on days 7, 14, and 21 yielded significantly lower t_20_ compared to day 0, but there was no significant difference between them. In terms of t_80_, days 14 and 21 had significantly lower measurements compared to days 0 and 7, but there was no difference between each of the first or last two days. Negative temporal bias on day 0 was indicatory of delayed acoustic emissions (Figure 6D). The bias was progressively eliminated towards day 21, due to the more uniform distribution of these emissions over time (Figure 6B). Harmonic amplitude decreased over time compared to the broadband floor (Figure 6E). This was reflected on the cavitation doses (Figure 6F). Whereas harmonic cavitation dose based on harmonics decreased over time (slope: −10 mV/d), ultraharmonic and inertial cavitation doses rose over time (slope: 0.46 mV/d and 0.8 mV/d, respectively).

Taken together, these results demonstrate that MBs get progressively less stable under therapeutic exposure *in vitro* over time. Given the limited variation in the size distribution for 3 weeks post-activation (Figure 5D), it is unlikely that changes in MB size drove this transition. It is likely that the lipid content is modified during storage, due to either ambient pressure or ambient temperature variations [17]. Surfactant shedding may change the total amount or the DSPC:DSPE-PEG2K ratio in the MB membrane [12,67]. According to our simulation results, a decrease in the lipid molar ratio would lead to MBs more prone to fragmentation (Figure 3C). A possible explanation is that DSPC is naturally expelled out of the MB shell in the examined time scale, possibly due to its charge and MB zeta-potential [68–70]. If the expulsion rate of the neutral emulsifier DSPE-PEG2K was lower than the respective rate of DSPC, the DSPC:DSPE-PEG2K molar ratio would effectively decrease over time. This would lead to MBs with decreased compression modulus and, most importantly, viscosity (Figure 3D). Apart from lipid shedding, lipid degradation and peroxidation may influence the shell properties over time, especially given the gas exchange between the activated vial and atmospheric air. However, this remains a hypothesis that will be tested in future work, possibly using fluorescently-tagged lipids [71,72]. Finally, shell modifications would change the resonance frequency of both isolated MBs [73] and MB populations [74,75], thereby affecting their fragmentation threshold [76,77].

### *Microbubble stability* in vivo

**FIGURE 7 |:**
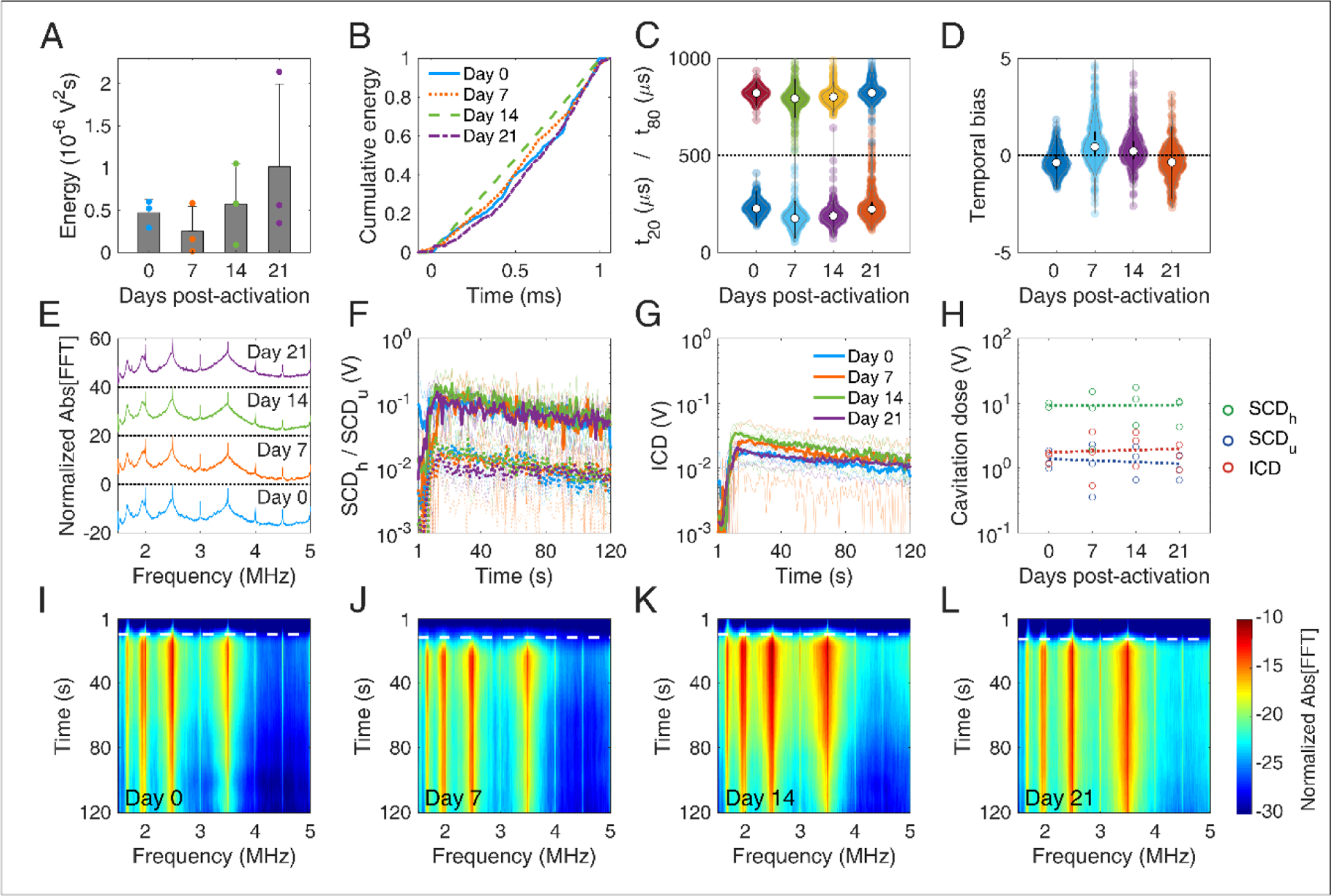
*In vivo* microbubble stability over time. (A) Mean acoustic energy emitted per mouse over time post-activation. (B) Mean cumulative energy evolution (n=10) for mice treated with focused ultrasound on day 0 (blue straight line), day 7 (dotted orange line), day 14 (dashed green line), and day 21 (dotted-dashed purples line) post-activation. (C) Temporal constants t_20_ (t < 500 μs) and t_80_ (t > 500 μs) over time post-activation. Temporal constants are plotted for each pulse and for each mouse (n=360) on a given time point. (D) Temporal bias over time. Temporal bias is plotted for each pulse and each mouse (n=360) on a given time point. (E) Normalized spectra averaged across pulses (n=10). (F) Temporal evolution of harmonic (straight lines) and ultraharmonic (dashed lines) stable cavitation levels over the course of a treatment session (t = 120s), averaged across mice (n=3). Transparent lines indicate the evolution of harmonic cavitation levels for each mouse. (G) Temporal evolution of inertial cavitation levels over the course of a treatment session (t = 120s), averaged across mice (n=3). Transparent lines indicate the evolution of inertial cavitation levels for each mouse. (H) Stable harmonic (green circles), stable ultraharmonic (blue circles), and inertial (red circles) cavitation doses over time. A linear fit was performed on each dose (dashed lines) to investigate the average effect of storage time on cavitation dose *in vivo*. (I)-(L) Spectrograms for FUS treatments on days 0, 7, 14, and 21 post-activation. Dashed white lines indicate the time point of MB entrance into the focal volume.

MBs with reduced stability during therapeutic pulses *in vitro* were expected to have similar but not identical behavior *in vivo*, due to the different boundary conditions [78–81]. Despite the large variation of emitted energy per pulse in each mouse, the total energy emitted during the 2-min FUS treatment was not significantly different across days (p = 0.46, n = 3 mice per day; Figure 7A). The average energy initially decreased at day 7, but then increased on average until day 21. We observed similar temporal distributions of the cumulative energy across days (Figure 7B). Temporal constants presented a wide deviation across all the pulses per day (Figure 7C). When examining the average constants per mouse, t_20_ was not found significantly different across days (p = 0.06, n = 3 mice per day) and t_80_ was marginally different (p = 0.02; only statistical difference was observed between day 7 and 21). Both constants had a similar general trend, initially decreasing on day 7 and then increasing until day 21. In other words, MBs appeared more stable during sonication on day 21, compared to days 7 and 14, and similarly stable compared to day 0. The inverse trend was observed in the temporal bias (p = 0.03), initially increasing above 0 (i.e., early emission bias) and then decreasing below 0 on day 21 (i.e., late emission bias). A possible explanation is experimental variations on day 21, e.g. injection of moderately higher MB concentration. Alternatively, MB response under confinement within the microvasculature *in vivo* is expected to be different compared to relatively unconfined oscillations occurring in the *in vitro* experiment [78,82]. However, this hypothesis should be tested in future work with variable confinement scales, for example using elastic tubes of different diameters on the micrometer scale [81].

Average spectra were qualitatively similar across days (Figure 7E). Harmonics and broadband signal had similar fine structure and relative amplitudes. We detected the Doppler shift from moving MBs as an asymmetric broadening of the harmonics towards lower frequencies, especially in the 4^th^ harmonic (i.e., 2 MHz). This effect has been observed before *in vitro* [62,83] and *in vivo* [84], and was also detected in the *in vitro* experiment presented here (Figure 6E). Stable (Figure 7F) and inertial (Figure 7G) cavitation doses rose upon MB entrance into the focus and were sustained throughout the 2-min sonication, albeit at a diminishing trend due to MB clearance from the bloodstream. Despite the large variation of cavitation doses during treatment, the total cavitation doses for each mouse had non-significant variation over storage time (p = 0.89 for SCD_h_, p = 0.92 for SCD_u_, and p = 0.71 for ICD; Figure 7H). Linear regression was performed taking into account all data points per dose (n = 3 mice per day, i.e. total of n = 12 data points), to identify potential trends over time. Harmonic stable and inertial cavitation doses moderately increased (slope 3.5 mV/d and 10 mV/d), while ultraharmonic stable cavitation dose decreased on average over time (slope −10 mV/d). The increase of inertial cavitation was evident in the spectrograms of FUS treatments for day 0 (Figure 7I), day 7 (Figure 7J), day 14 (Figure 7K) and day 21 (Figure 7L). Normalized broadband signal increased over time, especially for frequencies higher than 3.5 MHz (Figures 7I–L). The broadband emissions were sustained throughout treatment on day 21, despite their relatively lower amplitude compared to previous time points (Figures 7G and 7L).

*In vivo* data were in general agreement with the *in vitro* results (Figure 6). On average, inertial cavitation response increased with storage time (Figures 6F, 7H, 7I–L). Interestingly, despite the initial decrease of temporal stability, as indicated the t_20_ and t_80_ reduction (Figure 6C and 7C), the *in vivo* response rebounded and appeared higher on day 21 (Figure 7C). This may be due to the increased persistence of broadband emissions during the entire treatment (Figure 7L). The *in vivo* environment is different compared to the *in vitro* conditions, in terms of temperature (37 °C vs. 20 °C), host liquid viscosity (i.e. blood vs. water), blood flow rates, etc. Therefore, simulation results (Figure 3) may not apply directly *in vivo*, since many of the assumptions are violated. Importantly, spatial confinement of MBs within the microvasculature significantly affects their response and longevity [23,78,80,85]. Due to the low MB concentration used here (10^7^ MBs/ml or ~ 5× the clinical imaging dose), *in vivo* experiments were more prone to sampling errors due to the minute volumes required for intravenous injections into mice. Therefore, intravascular MB density may be different compared to the phantom channel, and may also differ across mice. This would affect the bubble-bubble interactions and the resulting acoustic emissions [74,75].

### Blood-brain barrier opening stability

**FIGURE 8 |:**
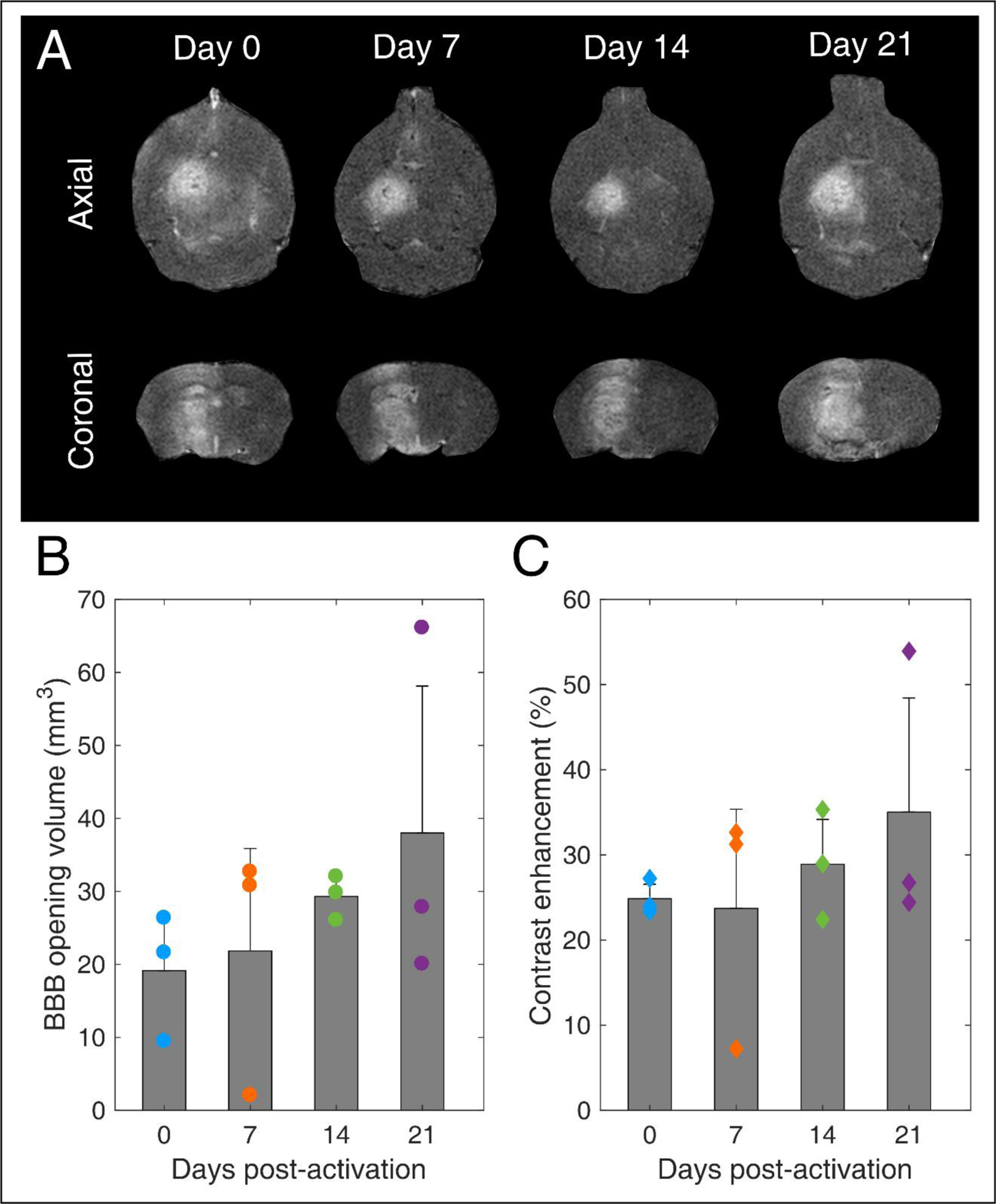
Blood-brain barrier opening over time. (A) Contrast-enhanced T1-weighted MRI axial (upper row) and coronal (lower row) scans for mice treated with FUS on day 0, 7, 14, and 21 after microbubble activation. (B) BBB opening volume over time. (C) Contrast enhancement over time. Gray bars indicate average values and error bars indicate standard deviation (n=3 mice).

Our main hypothesis in this study was that BBB opening efficiency is not affected by the MB storage time. T1-weighted contrast-enhanced MRI scans confirmed BBB opening the targeted structure in every treated mouse (Figure 8A). BBB opening volume was not significantly different across days (p = 0.49, n = 3 mice per day). However, the average volume increased over time. Specifically, it was measured as 19.1 ± 7.1 mm^3^, 21.8 ± 14 mm^3^, 29.3 ± 2.5 mm^3^, and 38 ± 20.1 mm^3^ on day 0, 7, 14, and 21, respectively. Similar effects were observed in terms of contrast enhancement. On average, there was no significant difference (p = 0.63, n = 3 mice per day). Yet, there was an increasing trend over time, with measured enhancements being 24.9 ± 1.7 %, 23.7 ± 11.7 %, 28.9 ± 5.3 %, and 35 ± 13.4 % on day 0, 7, 14, and 21, respectively.

It is well established that the MB response dictates both BBB opening volume and contrast enhancement [22,63,86,87]. Despite the non-significant average differences, the increasing trends can be explained in the light of reduced stability during exposure and increased broadband response over time (Figures 6F, 7H, 7I–7L). Broadband emissions are typically associated with existence of inertial cavitation [88]. Inertial MB collapses trigger jet formation and exert excessive stresses on the endothelial cells of vascular walls [89], thereby compromising safety [90]. Nevertheless, the relative amplitude of harmonic over broadband signals suggests that stable cavitation was the dominant mode both *in vitro* and *in vivo* with these treatment conditions (Table 2) at every time point (Figures 6F and 7H).

Our findings confirmed our initial hypothesis that long-term storage of activated MBs has no significant effect on BBB opening efficiency (Figure 7B and 7C). Currently, MBs are typically used once immediately after activation. We show here that this is not necessary, since MBs can be used multiple times following activation for up to 3 weeks post-activation without losing their therapeutic efficacy. This observation is likely to reduce the cost of both pre-clinical and clinical applications, provided that sterility is ensured throughout the storage period.

More importantly, the majority of the MBs used for therapeutic applications were originally designed and manufactured for contrast-enhanced ultrasound imaging applications. This study highlights the need for purpose-built MBs that are tailored to the intended therapeutic application, for example FUS-mediated BBB opening. Microbubble shell constitution affects the cavitation response of MBs exposed to therapeutic ultrasound pulses (Fig. 4). Although contrast agents such as Definity® or SonoVue® are optimal in providing contrast when exposed to microsecond-long imaging pulses, future therapeutic MBs should present enhanced temporal stability during low-frequency millisecond-long exposure (Fig. 7), to avoid compromising safety. The stability metrics provided in this study (Figs. 2, 4, 6, 7) may aid in the characterization of future MB formulations designed for therapeutic applications.

## CONCLUSIONS

In this study, we evaluated the temporal stability of lipid-shelled MBs during therapeutic ultrasound exposure. Simulations showed that the stiffness and viscosity of the MB shell influences the MB oscillation dynamics. We found that viscosity is the parameter dominating the fragmentation pressure at therapeutically-relevant insonation parameters. A DSPC:DSPE-PEG2K molar ratio of 9:1 was more stable experimentally compared to other shell configurations. MB concentration decreased over storage time, with a decay constant of 0.02 d^−1^. However, there was limited change in the mean and maximum radii of the MB population (< 10% variation). Storage time decreased the *in vitro* MB stability, decreasing stable cavitation response and promoting inertial cavitation over time. Similar response was observed *in vivo*, where we detected sustained inertial cavitation during therapeutic pulses only on day 21 post-activation. BBB opening volume and contrast enhancement were not significantly different across the tested time points, yet both followed an increasing trend. Our findings may be useful in understanding MB dynamics under therapeutic exposure and prove that repeated treatments using stored MBs are possible for both pre-clinical and clinical applications. Finally, this study highlights the need for MBs tailored to therapeutic applications and provides tools for assessing MB stability in the ultrasound therapy regime.

## Figures and Tables

**Table 1 |:** Parameters for numerical simulations of microbubble stability

Symbol	Description	Value
R_0_	Microbubble equilibrium radius	1.2 × 10^−6^ m
R_buckling_	Microbubble buckling radius	1.2 × 10^−6^ m
ρ_1_	Liquid density (water)	10^3^ kg/m^3^
μ	Liquid viscosity (water)	10^−3^ Pa × s
P_0_	Ambient hydrostatic pressure	10^5^ Pa
c	Speed of sound	1.48 × 10^3^ m/s
κ	Polytropic gas coefficient	1.095
σ_water_	Water surface tension	0.073 N/m
σ_break-up_	Break-up surface tension	0.2 N/m
ε	Shell thickness	10^−9^ m
χ	Compression modulus	0.042 – 0.116 N/m
κ_S_	Surface dilatational viscosity	4 - 6.5 × 10^−10^ Pa × m × s
f_c_	Center frequency	0.5 × 10^6^ Hz
P	Peak-negative pressure	50-350 × 10^3^ Pa

**Table 2 |:** Acoustic parameters used in both *in vitro* and *in vivo* experiments

Parameter	Value
Center frequency	0.5 MHz
Peak-negative pressure	300 kPa
Pulse length	1 ms or 500 cycles
Pulse repetition frequency	1 Hz
Sonication duration	2 min or 120 pulses
Microbubble dose	10^7^ MBs/ml
